# An echographic study of follicular growth during induced estrus in female Azawak zebu in Niger

**DOI:** 10.1007/s11250-015-0871-y

**Published:** 2015-06-21

**Authors:** Mahamadou Moussa Garba, Moumouni Issa, Hamani Marichatou, Christian Hanzen

**Affiliations:** Faculty of Agronomy, University Abdou Moumouni, Niamey, Niger; Faculty of Veterinary Medicine, Theriogenology Department of Animal Production, University of Liege, B42 Sart Tilman, 4000 Liege, Belgium

**Keywords:** *Bos indicus*, Echography, Estrus, Ovulation, PGF_2α_, Progesterone, ECG

## Abstract

An echographic study of follicular growth up to ovulation was carried out on 42 lactating Azawak cows (*Bos indicus*) after estrus induction by means of a PGF_2α_ or a procedure involving the administration of progesterone vaginally (PRID® DELTA: progesterone-releasing intravaginal device) for a 10-day period and the injection of a PGF_2α_ and an ECG (400 and 800 IU) on withdrawal. All the animals were inseminated 12 and 24 h after the onset of estrus. The percentage of estrus induced was not significantly different between the two groups of animals (81 %). The average time delay before the onset of estrus was significantly longer after injection of a PGF_2α_ (84.8 ± 26.0 h) than after withdrawal of the PRID® (59.2 ± 5.8 h). The average duration of the estrus was significantly shorter after its induction by PGF_2α_ (12.6 ± 2.6 h) than after induction by progesterone (22.9 ± 2.7 h). There was not a significant difference in the interval between the beginning of estrus and ovulation in animals treated by PGF_2α_ (30.3 h) and progesterone (28.4 h). Injection of a PGF_2α_ was accompanied by a significantly lower rate of gestation than that obtained after treatment of animals by progesterone (31.2 vs 54.5 %). These results confirm the necessity to adapt an insemination policy to hormonal treatment for estrus induction in *B. indicus*.

## Introduction

Reduced reproductive performance is one of the characteristics of cattle-breeding in Africa. This is illustrated by an increase in the age of the first calving (45 to 60 months) and the interval between calvings which is often longer than 15 months (Denis and Thiongane 1973; Sokouri et al. 2010). The long interval between calving and first insemination (voluntary waiting period) is the main contributing factor in both cases. Such situation limits the genetic progress and profitability of *Bos indicus* breeding in particular. In addition, given the breeding conditions and heat detection in the cows, the extensive use of artificial insemination can only be achieved through hormonal programs aimed at inducing and synchronizing heat.

Various procedures for heat induction were used in *B. indicus*. They consist of the individual or associated use of PGF_2α_, progestogens (progesterone or norgestomet), and equine chorionic gonadotropin (ECG) (Baruselli et al. 2004; Voh et al. 2004; Wéré et al. 2012a, b). According to studies carried out, the gestation rates are 35.7 % in the Gobra cow (Mbaye and Ndiaye 1993), 57.6 % in the Nellore cow (Sa’ Filho et al. 2009), 66.6 % in the Wadara cow (Vounparet et al. 2014), between 21 and 42 % in the Azawak cow (Zongo et al. 2001, 2012), and between 42 and 45 % in the Goudali cow (Wéré et al. 2012a, b; Zongo et al. 2012). Different echographic studies have made it possible to identify the interval between the beginning of heat and ovulation in *B. indicus* which is 25.6 ± 2.8 h in the Gobra zebu in Chad (Vounparet et al. 2014), 25.8 h in the Ethiopian zebu (Mukasa-Mugerwa and Mattoni 1988), 26.6 ± 0.4 h in Nellore female zebus after natural or induced estrus, and between 22 and 26 h in Goudali zebu cows (Wéré et al. 2012a, b).

The aims of the study were to (1) describe, by means of echography, the characteristics of follicular growth and ovulation in Azawak cows after estrus induction by PGF_2α_, or progestogens; (2) compare the pregnancy rate after insemination of treated animals; and (3) specify the optimum time for insemination.

## Material and methods

The study was carried out at the Toukounous station, located 200 km to the North of Niamey (Niger) in February 2014 with an average temperature of 20 °C. It is a dairy station where the main system of breeding is natural mating. The animals have been divided into herds based on age, sex, and physiological condition. They live on the natural pasture land of the station. Only lactating cows receive supplementary cotton seed meal. It involved 42 lactating cows of the Azawak indigenous breed. After a clinical examination carried out by manual palpation and echography (echography model KX5200V, a linear transrectal probe of 6.5 MHz), these cows were divided into three experimental groups. The cows of group 1 (*n* = 20) presenting with a corpus luteum during the examination were treated by means of a 25-mg PGF_2α_ injection (Dinoprost®). This injection was carried out 12.0 ± 3.3 days after the previous heat. The cows of group 2 (*n* = 13) and group 3 (*n* = 9) which did not have a corpus luteum were treated by means of an intravaginal coil (PRID® DELTA progesterone-releasing intravaginal device) containing 1.5 g of progesterone. On the 8th day of treatment, an injection of 25 mg of PGF_2α_ was administered to make certain of the luteolysis of the corpus luteum that was not identified or that was insufficiently developed during the first examination. At the end of the treatment, the cows of groups 2 and 3 were treated by IM injection, with doses of 400 and 800 IU of ECG (equine chorionic gonadotropin Folligon®), respectively. The body condition score (BCS) of the cows was determined on a scale of 1 to 5, on the one hand, by palpation of the lumbar region of the animal and, on the other hand, by observation of the general appearance of the animal (Vounparet et al. 2014) during the application of the treatments.

An echographic examination of the ovaries was carried out twice a day on all the cows up to confirmation of ovulation. During each examination, the diameter of the largest follicle identified as an anechogenic area was measured and follicles with a diameter higher than 4 mm present on the two ovaries were counted. Ovulation was indirectly confirmed by the disappearance of the largest follicle or by the sudden reduction in the size of its diameter during examination. The signs of heat (passive covering, mucous secretion) were identified by twice-daily visual observation (8 and 18 h) carried out during the 7 days following the treatment. Two artificial inseminations by means of frozen sperm were carried out 12 and 14 h after the detection of the estrus in 16 and 11 of the females of groups 1 and 2, respectively. A gestation observation was carried out by manual palpation of the cows 60 days later.

The treatments were compared by means of the following parameters: interval (h) between the treatment (injection of PGF_2α_ or removal of the coil) and the beginning of estrus (TE), and ovulation (TO), duration of estrus (h) (DE), number of follicles with a diameter higher than 4 mm determined every 12 h during the 6 days following termination of the treatment (NF), and the diameter of the ovulatory follicle (mm) (DF). The number of follicles with a diameter higher than 4 mm (NF) was determined for the 42 cows by means of echographic examination carried out every 12 h during the 6 days following termination of the treatment. The diameter of the ovulatory follicle (mm) refers to the largest diameter identified by echography before its disappearance. This diameter has been calculated for the 34 cows for which an estrus was detected after treatment.

Statistical analyses were carried out using Statistical Analysis 95 System (SAS, 2001). The data measured was first subjected to a normality test. A variance analysis was carried out in accordance with the GLM (general linear models procedure), which is a procedure to determine the effect of the treatment on the diameter of the follicle. The time delay for the estrus, the duration of the estrus, the time delay for the ovulation, data whose distribution does not follow a normal law were submitted to the “Kruskal-Wallis” non-parametric test. The differences were considered as significant when they reached a threshold of *P* < 0.05.

## Results

The average of age, number of calvings, BCS, and number of postpartum days, respectively, were equal to 9.5 ± 4 years, 4.9 ± 3.2, 3.6 ± 0.4, and 299 ± 190.0 days. There was no significant difference (*P* ˃ 0.05) observed between the three experimental groups (Table 1).

**Table 1:** General characteristics of females in the experimental groups

	Number	Treatment	Age	NC	Score	DPP
Group 1	20	PGF_2α_	10.2 ± 3.8	5.8 ± 3.1	3.5 ± 0.4	276.6 ± 155.0
Group 2	13	P-PGF_2α_-ECG 400 IU	8.5 ± 4.7	4.1 ± 3.8	3.5 ± 0.5	325.2 ± 221.7
Group 3	9	P-PGF_2α_-ECG 800 IU	9.6 ± 3.6	4.2 ± 2.5	3.8 ± 0.2	312.4 ± 230.7
Average	42		9.5 ± 4.0	4.9 ± 3.2	3.6 ± 0.4	299.3 ± 190.8
*P*			0.4	0.2	0.09	0.6

*P* PRID®, *NC* number of calvings, *BCS* body condition score, *DPP* days postpartum

The average for induced estrus was from 81 % (34/42). No significant difference was observed between hormonal treatments.

The average diameter of the corpus luteum of the cows whose estrus was induced by PGF_2α_ determined by ultrasound was 20.0 ± 2.7, 15.7 ± 2.2, 13.5 ± 2.8, 10.5 ± 5.3, and 10.4 ± 2.3 after 0, 12, 24, 36, and 48 h, respectively, following injection.

The parameters studied on the cows whose estrus was observed presented significant differences between the treatments carried out (Table 2).

**Table 2:** The compared effect of treatments on the parameters studied among the 34 cows that presented with an estrus

Factors	Number	Estrus (%)	TE (h)	DE (h)	TO (h)	NF	DF
Group 1	20	81	84.8^b^ ± 26.0	12.6^b^ ± 2.6	112.9^b^ ± 22,6	4.2 ± 2.7	10.7 ± 2.6
Group 2	13	84	60.3^a^ ± 20.7	22.6^a^ ± 2.8	88.5^a^ ± 19.2	3.4 ± 1.44331	9.5 ± 2.3
Group 3	9	78	56.2^a^ ± 10.5	23.7^a^ ± 2.5	85.5^a^ ± 13.7	4.7 ± 1.6	9.0 ± 2.2
Groups 2 and 3	22	81	59.2^a^ ± 5.8	22.8^a^ ± 2.7	87.5^a^ ± 7.2	4.0 ± 1.6	9.3 ± 2.3
Average	42	81	72.4 ± 25.7	17.4 ± 5.8	100.6 ± 23.4	4.1 ± 2.3	9.9 ± 2.5
*P*			0.01	˂0.0001	0.003	˃˃0.05	˃˃0.05

The figures followed by different letters (a, b) in the same column indicate a significant difference in the range of *P* ˂ 0.05

*N* number of cows, *TE* interval (h) between the injection of PGF_2α_ or removal of the coil and the beginning of estrus, *TO* interval (h) between the injection of PGF_2α_ or removal of the coil and ovulation, *DE* duration of the estrus (h), *NF* number of follicles with a diameter larger than 4 mm, *DF* diameter of the ovulatory follicle (mm)

The average time between the injection of PGF_2α_ or PRID® removal and the detection of the estrus (TE) appeared to be significantly longer (*P* = 0.01) among the females of group 1 (84.8 ± 26.0 h) than in those of groups 2 (60.3 ± 20.7 h) and 3 (56.2 ± 10.5 h). Conversely, the average duration of the estrus (DE) was significantly shorter (*P* ˂ 0. 0001) among the females of group 1 (12.6 ± 2.6 h) than among those of groups 2 (22.6 ± 2.8 h) or 3 (23.7 ± 2.5 h). In the same way, it was significantly shorter in cows treated by means of PGF_2α_, (group 1, 12.6 ± 2.6 h) than among those treated by means of progesterone (groups 2 and 3, 22.8 ± 2.7 h). After injection of PGF_2α_, the percentage of cows observed to be in heat after 3, 4, 5, 6, and 7 days were 38, 75, 88, 94, and 100 %, respectively. These percentages were 86 and 100 %, respectively, 2 and 3 days after removal of the PRID®.

The interval between treatment and ovulation (TO) was significantly longer (*P* = 0,006) for the animals of group 1 (112.8 ± 4.9 h) than for those of groups 2 (88.4 ± 9.5 h) and 3 (85.5 ± 13.7 h). By comparison, the interval between the beginning of estrus and the moment of ovulation was not significantly different (*P* = 0.25) in animals treated with PGF_2α_ (30.3 ± 9.3 h) in relation to those treated by 400 IU (28.0 ± 4.9 h) and 800 IU (29.2 ± 6.9 h) ECG. In addition, this number was not significantly different in animals treated with PGF_2α_ (30.3 ± 9.3 h) compared to those treated by means of progesterone (28.4 ± 5.3 h). For the 8 animals not presenting with estrus, 4 of the groups 2 (*n* = 2), and 3 (*n* = 2) ovulated 102 ± 8.4 h after treatment.

The average number of follicles with a diameter higher than 4 mm (NF) observed by ultrasound during the 144 h following the treatment was not significantly different among the animals treated with PGF_2α_, (4.2) in relation to those treated with 400 IU (3.4) and 800 IU (4.7) ECG. In the same way, this number was not significantly different in animals treated with PGF_2α_, (4.2) compared with those treated by means of progesterone (4.0).

The diameter of the ovulatory follicle observed by ultrasound during the 108 h following the treatment of the animals treated with PGF_2α_ (10.7) was not significantly different from that observed during the 84 h following treatment of the animals treated with 400 IU (9.5) and 800 IU (9.0) ECG. In the same way, this diameter was not significantly different in the animals treated with PGF_2α_, (10.7) in relation to those treated by means of progesterone (9.3). The average daily growth of the ovulatory follicle was 1.4 ± 0.7 and 1.6 ± 0.8 mm; 1.3 ± 0.7 and 1.3 ± 0.5 mm, respectively, in groups 1 to 3.

The average percentage for gestation of the 27 cows that were inseminated (groups 1 and 2) was 40.7. It was significantly higher in cows treated with a PRID® (54.5 %) than with a PGF_2α_ (31.2 %).

## Discussion

Used animals are relatively old (9.5 ± 4.0 years on average). The experiment was done at the Sahelian Experimental Station of Toukounous that is the only station which has pure selected Azawak cattle, animals have index cards of follow-up. The use of cows 8 to 10 years old is guided by the fact that they are animals available and intended for the experiments at the time of the study.

Whether induced by a PGF_2α_ injection associated or not with a progestogen, heat was observed in 81 % of cases. This percentage is comparable to those observed (66.6 to 90.5 %) in zebus treated by means of a single injection (Rekwot et al. 1999) or repeated at 11-day intervals with a PGF_2α_ injection (76.5 to 100 %) (Cissé 1993; Vounparet et al. 2014) or by means of a progestogen (57.1 to 100 %) (Issa et al. 2013; Sa’ Filho et al. 2009; Vounparet et al. 2014; Wéré et al. 2012a, b; Zongo et al. 2012). Similar values were observed among *Bos taurus* females treated with a progestogen (Hanzen and Laurent 1991) or a PGF_2α_ injection (Hanzen et al. 2003).

The average interval between treatment and the appearance of estrus appeared to be significantly longer after a PGF_2α_ injection (84.8 ± 26.0 h) than after the removal of the PRID® (59.2 ± 5.8 h). They seem to be significantly longer than those observed after treatment of *B. indicus* cows by means of a norgestomet implant. Intervals between 32.0 ± 1.1 h (Tegegne et al. 1989), 32.7 ± 4.7 h (Wéré et al. 2012a, b), and 33.1 ± 4.3 h (Zongo et al. 2012) have been observed. Several factors can affect the appearance on the estrus, in particular, the method of induction, the age of the corpus luteum at the moment of treating the animal by means of a PGF_2α_, the postpartum, and the age of animals.

The average duration of estrus was shorter after its induction by means of a PGF_2α_ injection (12.6 ± 2.6 h) than by a progestogen (22.9 ± 2.7 h). A similar duration of estrus induced by a progestogen was also observed (Marichatou et al. 2010). The duration of estrus observed after a PGF_2α_ injection is comparable to those observed in *B. taurus* and recorded as being between 11.8 ± 4.4 h (Voh et al. 2004) and 13.6 ± 2 h (Wéré et al. 2012a, b).

The distribution of the intervals between treatment and the appearance of estrus was also higher. The follicular status at the moment of treating the animal by means of a PGF_2α_ injection or a progestogen explains the differences observed. In *B. taurus*, estrus occurs later after administering a PGF_2α_ injection between days 10 and 15 of the cycle than if the injection is administered between days 5 and 9 (Hanzen et al. 2003). This effect of the stage of the cycle is due to a different follicular population at the moment of injection. The interval between the PGF_2α_ injection and estrus will be shorter if it coincides with the presence of a dominant follicle. It will be longer when the dominant follicle is in a regression phase because, in this case, the time delay for obtaining a new pre-ovulatory follicle and therefore an estrus is prolonged (Lane et al. 2008). Thus, in the case of *B. taurus*, the estrus occurs later after administration of the PGF_2α_ injection between days 10 and 15 of the cycle than if the injection is carried out between the 5th and 9th days of the cycle (Beal 1996).

The distribution of returns to heat is comparable to that observed in *B. taurus* (86 % after the 5 first days following the injection) (Hanzen et al. 2003). A treatment based on progestogens contributes to reducing the timescale and the distribution of returns to estrus especially if it is accompanied by a PGF_2α_ injection before the end of the treatment. The progestogen induces atresia of the eventual dominant follicle present because it causes a reduction in the synthesis of LH. The result is the appearance of a new wave of follicular growth 4 to 5 days later (Bo et al. 1995; Rhodes et al. 2002). Moreover, if at the moment of progestogen treatment the animal was in metestrus, a PGF_2α_ injection 1 to 2 days before stopping treatment is advised especially if the progestogens have been administered vaginally. This treatment has the effect of removing the inhibiting effect of endogenous progesterone and ensuring an optimal release of hypophyseal hormones (Mialot et al. 1998).

This observation takes on a practical importance because it conditions the optimal moment for insemination in *B. indicus* after detection of an estrus or systematically 48 h (Sa’ Filho et al. 2009; Wéré et al. 2012a, b) or 48 and 72 h (Vounparet et al. 2014; Zongo et al. 2012) after the removal of the implant or an intravaginal device. In case of induction of the estrus by means of a PGF_2α_ injection, it would appear preferable to carry out a double insemination at 72 and 96 h, these two inseminations having been carried out some 12 h earlier in heifers (Lucy et al. 2004; Stevenson et al. 1996).

The ovulation was indirectly confirmed by the disappearance of the preovulatory follicle between two successive ultrasound examinations or by the sudden reduction in the size of its diameter. The interval between estrus and ovulation was not significantly different in animals treated by means of PGF_2α_ injection (30.3 h) and progestogens (28.4 h). These values are comparable to those observed in *B. taurus* and known to be between 27.6 ± 5.4 h (Walker et al. 1996) and 30.0 ± 5.1 h (Roelofs et al. 2005).

The treatment of animals by means of a PGF_2α_ injection is accompanied by a significantly lower gestation rate (31.2 %) to that obtained after use of a procedure based on progestogens associated with a PGF_2α_ injection and ECG (54.5 %). Such differences have also been observed by other authors. Therefore, pregnancy rates were 29.4 and 40.0 %, respectively, after treatment of Wadara zebus (Vounparet et al. 2014) and Maure zebus (Cissé 1993) by double injection of PGF_2α_, at 11-day intervals. A recent study (Moussa et al. 2014) observed that there were pregnancy rates of between 21.6 and 66.6 % (average value: 46.6 %) after treatment of the different breeds of *B. indicus* by means of progesterone.

## Conclusion

The use of hormonal protocols (PGF_2α_ injection and/or progestogens associated with ECG) in the Azawak cow (*B. indicus*) for estrus synchronization improves reproductive performance. We recommend that, in Niger, animals treated with progestogens be inseminated earlier than those treated with PGF_2α_.
